# Supplementary material for: Metabolomic Signatures of Alzheimer’s Disease Indicate Brain Region-Specific Neurodegenerative Progression
Source: Int J Mol Sci. 2023 Sep 30;24(19):14769. doi: 10.3390/ijms241914769 (PMC10573054; doi:10.3390/ijms241914769)
Supplement: Supplementary file 1 [file ijms-24-14769-s001.zip › ijms-2615436-supplementary.pdf]

	Brain Regions							
**Metabolite**	**BA 9**	**BA 17**	**BA 22**	**BA 24**	**BA 40**	**DN**	**HPC**	**PB**
**Acetylcholine**	↑		↓	↓	↓	↓		
**2-Amino-3-Phosphonoprionic Acid**	↑	↓	↓		↓		↓	
**Arginine**		↓			↓			
**Citric Acid**	↑		↑	↑		↑	↑	↑
**Creatine**	↓	↑	↑				↑	
**Ethanol**	↓	↑	↓	↑	↑	↑		
**Gamma-Aminobutyric Acid (GABA)**	↑	↑	↑	↑	↑		↑	
**Glycyl-Glycine**	↑	↓		↓		↓		↓
**Glycerol**	↑	↑	↓				↑	↑
**Glucose**	↑	↓		↓	↓			↓
**Isoleucine**		↓	↓	↓	↓	↓	↓	↓
**Leucine**	↓	↓	↓	↓	↓		↓	↓
**Malonic Acid**	↑	↑	↑	↑		↑	↑	
**N-acetylaspartate (NAA)**	↑	↑	↑	↑	↑		↑	↑
**Phenylalanine**	↑	↓	↓	↓	↓		↓	↓
**Phosphorylcholine**	↑	↓	↓	↓	↓		↑	
**Serine**		↓	↓		↓	↓	↓	
**Tyrosine**	↑	↓	↓		↓		↓	↓
**Valine**	↑	↓	↓	↓	↓		↓	↓

Supplemental table S1: Summary of the most notable significant metabolites for BA9, BA17, BA22, BA24, BA40, DN, HPC, and PD and their regulation (↑ upregulated; ↓ downregulated).
